# Bis(μ-*N*-benzyl-*N*-methyl­dithio­carbamato)-1:2κ^3^ *S*,*S*′:*S*′;1:2κ^3^ *S*:*S*,*S*′-bis­[bis­(*N*-benzyl-*N*-methyl­dithio­carbamato-κ^2^ *S*,*S*′)thallium(III)]

**DOI:** 10.1107/S1600536808021004

**Published:** 2008-07-12

**Authors:** Corrado Rizzoli, Kuppukkannu Ramalingam, Nagarajan Alexander

**Affiliations:** a Dipartimento di Chimica Generale ed Inorganica, Chimica Analitica, Chimica Fisica, Viale G. P. Usberti 17/A, Universitá di Parma, I-43100 Parma, Italy; b Department of Chemistry, Annamalai University, Annamalainagar 608 002, Tamilnadu, India

## Abstract

The molecule of the dinuclear title compound, [Tl_2_(C_9_H_10_NS_2_)_6_], possesses a crystallographically imposed centre of symmetry. Each Tl^III^ atom is seven-coordinated by S atoms of four different dithio­carbamate anions in a distorted penta­gonal-bipyramidal coordination geometry. The crystal structure is stabilized by a C—H⋯S hydrogen-bond inter­action linking complex mol­ecules into chains running parallel to the *b* axis. Intramolecular C—H⋯S hydrogen bonds are also present.

## Related literature

For the crystal structures of Tl-dithio­carbamate complexes, see: Abrahamson *et al.* (1975 ▶); Burschka (1982 ▶); Casas *et al.* (1994 ▶); Griffin *et al.* (1980 ▶); Ivanov *et al.* (2006 ▶); Jennische *et al.* (1972 ▶); Kepert *et al.* (1978 ▶); Nilson & Hesse (1969 ▶); Pritzkow & Jennische (1975 ▶).
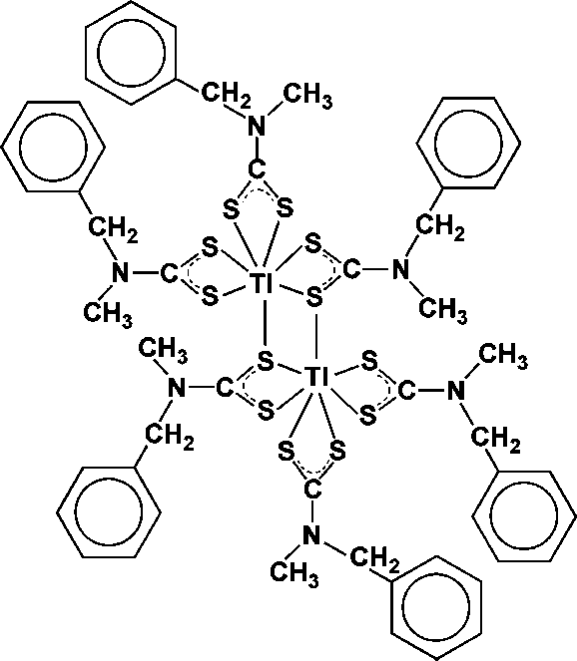


## Experimental

### 

#### Crystal data

[Tl_2_(C_9_H_10_NS_2_)_6_]
*M* *_r_* = 1586.57
Monoclinic, 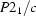

*a* = 13.3326 (9) Å
*b* = 9.9280 (6) Å
*c* = 24.1379 (16) Å
β = 98.539 (2)°
*V* = 3159.6 (4) Å^3^
*Z* = 2
Mo *K*α radiation
μ = 5.53 mm^−1^
*T* = 296 (2) K
0.27 × 0.24 × 0.23 mm

#### Data collection

Bruker SMART 1000 CCD diffractometer
Absorption correction: multi-scan (*SADABS*; Bruker, 1997 ▶) *T*_min_ = 0.248, *T*_max_ = 0.282
39462 measured reflections
8268 independent reflections
5534 reflections with *I* > 2σ(*I*)
*R*_int_ = 0.040

#### Refinement

*R*[*F* ^2^ > 2σ(*F* ^2^)] = 0.026
*wR*(*F* ^2^) = 0.044
*S* = 0.94
8268 reflections
334 parameters
H-atom parameters constrained
Δρ_max_ = 0.45 e Å^−3^
Δρ_min_ = −0.74 e Å^−3^

### 

Data collection: *SMART* (Bruker, 1997 ▶); cell refinement: *SAINT* (Bruker, 1997 ▶); data reduction: *SAINT*; program(s) used to solve structure: *SIR97* (Altomare *et al.*, 1999 ▶); program(s) used to refine structure: *SHELXL97* (Sheldrick, 2008 ▶); molecular graphics: *ORTEP-3 for Windows* (Farrugia, 1997 ▶) and *SCHAKAL* (Keller, 1997 ▶); software used to prepare material for publication: *SHELXL97* and *PARST95* (Nardelli, 1995 ▶).

## Supplementary Material

Crystal structure: contains datablocks global, I. DOI: 10.1107/S1600536808021004/at2589sup1.cif

Structure factors: contains datablocks I. DOI: 10.1107/S1600536808021004/at2589Isup2.hkl

Additional supplementary materials:  crystallographic information; 3D view; checkCIF report

## Figures and Tables

**Table:** 

Tl1—S1	2.9241 (8)
Tl1—S2	2.6210 (8)
Tl1—S3	3.0242 (8)
Tl1—S4	2.8736 (8)
Tl1—S5	2.7325 (9)
Tl1—S6	2.8109 (8)
Tl1—S3^i^	3.1605 (8)

**Table:** 

S1—Tl1—S3	78.22 (2)
S4—Tl1—S3	60.32 (2)
S5—Tl1—S4	76.80 (3)
S5—Tl1—S6	64.52 (3)
S6—Tl1—S1	82.94 (2)
S2—Tl1—S3^i^	163.05 (2)

Symmetry code: (i) 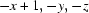.

**Table 2:** Hydrogen-bond geometry (Å, °)

*D*—H⋯*A*	*D*—H	H⋯*A*	*D*⋯*A*	*D*—H⋯*A*
C2—H2*B*⋯S1	0.97	2.51	3.030 (5)	113
C11—H11*A*⋯S3	0.97	2.51	3.009 (3)	112
C18—H18*C*⋯S4	0.96	2.51	3.008 (3)	112
C20—H20*B*⋯S5	0.97	2.46	3.012 (4)	116
C18—H18*A*⋯S1^ii^	0.96	2.87	3.654 (3)	140

Symmetry code: (ii) 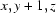.

**Fig. 1.**
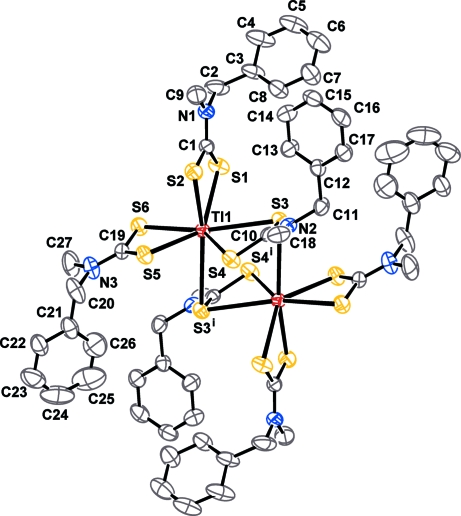
View of the title dinuclear complex molecule with displacement ellipsoids drawn at the 30% probability level. Hydrogen atoms are omitted for clarity [Symmetry code: (i) = 1 - x, -y, -z].

**Fig. 2.**
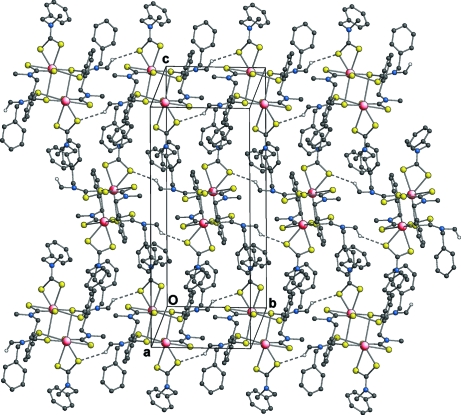
View of the crystal packing of the title compound. Intermolecular hydrogen bonds are shown as dashed lines. H atoms not involved in hydrogen bonds are omitted for clarity. Colour codes: Tl red, S yellow, N blue, C grey and H white.

## supplementary crystallographic information

### Comment

Thallium exhibits an interesting metallorganic chemistry with formal oxidation states of +1 and +3 and various coordination polyhedra. However, at the best of our knowledge, reports on the structural characterization of thallium dithiocarbamates are scarce. As part of our ongoing study on the chemistry of a series of new dithiocarbamato-containing metal derivatives, the dinuclear title complex was synthesized and its crystal structure is reported here.

The title compound (Fig. 1) possesses a crystallographically imposed centre of symmetry. Each thallium(III) metal centre exhibits seven-coordination provided by the sulfur atoms of four dithiocarbamate anions, two of which acting as bidentate chelating ligands and two as bidentate bridging ligands. The coordination geometry can be described as distorted pentagonal bipyramidal, with atoms S1, S3, S4, S5 and S6 defining the equatorial plane, and atoms S2 and S3^i^ occupying the apical positions [symmetry code: (i) 1 - *x*, -*y*, -*z*]. The Tl···Tl separation in the dinuclear complex molecule is 3.9221 (2) Å. The values of the Tl—S bond lengths involving the bidentate chelating ligands (Table 1) range from 2.6210 (8) to 2.9240 (10) Å, in agreement with those observed in other Tl-dithiocarbamato complexes (Burschka, 1982; Kepert *et al.*, 1978; Griffin *et al.*, 1980; Abrahamson *et al.*, 1975; Casas *et al.*, 1994). The Tl—S bond lengths involving the bridging S3 atom [3.0242 (8) and 3.1605 (8) Å] are consistent with the values reported in the literature for similar thallium(I) derivatives (Ivanov *et al.*, 2006; Jennische *et al.*, 1972; Pritzkow & Jennische, 1975; Nilson & Hesse, 1969). The C—S bond lengths within the dithiocarbamate ligands [mean value 1.714 (3) Å] fall in a rather narrow range of values suggesting a substantial delocalization of the double bond. The short values of the thioureide C—N distances [mean value 1.339 (4) Å] indicate that the electron density is delocalized over the NCS_2_ groups and these bonds have a partial double bond character. In the IR spectrum of the title compound, the important stretching mode characteristic of thioureide C—N bond occurs at 1475 cm^-1^. Thallium(III) possesses completely filled *d* orbitals whereas charge transfer (CT) is fully allowed and hence intense CT absorption is observed. Intraligand transitions of the dithiocarbamate anions are also observed along with a ligand-metal charge transfer (LMCT). The conformation of the dithiocarbamate anions is stabilized by intramolecular C—H···S hydrogen bonding interactions (Table 2). In the crystal structure (Fig. 2), dinuclear complex molecules are linked by an intermolecular C—H···S hydrogen bond (Table 2) into chains running parallel to the *b* axis.

### Experimental

Benzylmethylamine (6 mmol) and carbon disulfide (6 mmol) in ethanol (5 ml) were mixed under ice-cold conditions to obtain a yellow solution of *N*-benzyl-*N*-methyldithiocarbamic acid. An aqueous solution (2 ml) of TlF3 (2 mmol) was then added with constant stirring. The resulting precipitate was filtered off and dried in air. Single crystals of the title compound suitable for X-ray analysis were obtained by slow evaporation of an ethanol solution at room temperature.

### Crystal data

**Table:**

[Tl_2_(C_9_H_10_NS_2_)_6_]	*F*_000_ = 1560
*M**_r_* = 1586.57	*D*_x_ = 1.668 Mg m^−^^3^
Monoclinic, *P*2_1_/*c*	Mo *K*α radiation λ = 0.71073 Å
Hall symbol: -P 2ybc	Cell parameters from 6832 reflections
*a* = 13.3326 (9) Å	θ = 3.0–27.2º
*b* = 9.9280 (6) Å	µ = 5.53 mm^−^^1^
*c* = 24.1379 (16) Å	*T* = 296 (2) K
β = 98.539 (2)º	Block, yellow
*V* = 3159.6 (4) Å^3^	0.27 × 0.24 × 0.23 mm
*Z* = 2	

### Data collection

**Table:**

Bruker SMART 1000 CCD diffractometer	8268 independent reflections
Radiation source: fine-focus sealed tube	5534 reflections with *I* > 2σ(*I*)
Monochromator: graphite	*R*_int_ = 0.040
*T* = 296(2) K	θ_max_ = 29.8º
ω scans	θ_min_ = 1.5º
Absorption correction: multi-scan(SADABS; Bruker, 1997)	*h* = −18→18
*T*_min_ = 0.248, *T*_max_ = 0.282	*k* = −13→13
39462 measured reflections	*l* = −32→33

### Refinement

**Table:**

Refinement on *F*^2^	Secondary atom site location: difference Fourier map
Least-squares matrix: full	Hydrogen site location: inferred from neighbouring sites
*R*[*F*^2^ > 2σ(*F*^2^)] = 0.026	H-atom parameters constrained
*wR*(*F*^2^) = 0.044	*w* = 1/[σ^2^(*F*_o_^2^) + (0.0165*P*)^2^] where *P* = (*F*_o_^2^ + 2*F*_c_^2^)/3
*S* = 0.94	(Δ/σ)_max_ = 0.002
8268 reflections	Δρ_max_ = 0.45 e Å^−^^3^
334 parameters	Δρ_min_ = −0.74 e Å^−^^3^
Primary atom site location: structure-invariant direct methods	

### Special details

**Table:**

Experimental. All H atoms were placed in geometrically idealized positions and constrained to ride on their parent atoms, with C—H = 0.93–0.97 Å and *U*_iso_(H) = 1.2 *U*_eq_(C) or 1.5 *U*_eq_(C) for methyl H atoms.
Refinement. Refinement of *F*^2^ against ALL reflections. The weighted *R*-factor *wR* and goodness of fit *S* are based on *F*^2^, conventional *R*-factors *R* are based on *F*, with *F* set to zero for negative *F*^2^. The threshold expression of *F*^2^ > σ(*F*^2^) is used only for calculating *R*-factors(gt) *etc*. and is not relevant to the choice of reflections for refinement. *R*-factors based on *F*^2^ are statistically about twice as large as those based on *F*, and *R*- factors based on ALL data will be even larger.

### Fractional atomic coordinates and isotropic or equivalent isotropic displacement parameters (Å^2^)

**Table:**

	*x*	*y*	*z*	*U*_iso_*/*U*_eq_	
Tl1	0.556603 (8)	−0.057237 (10)	0.075989 (4)	0.04911 (4)	
S1	0.40839 (7)	−0.19940 (8)	0.13240 (4)	0.0762 (2)	
S2	0.54015 (7)	0.02867 (9)	0.17692 (3)	0.0734 (2)	
S3	0.36641 (6)	0.10572 (8)	0.04008 (3)	0.0615 (2)	
S4	0.57292 (6)	0.22115 (8)	0.04598 (4)	0.0722 (2)	
S5	0.75951 (6)	−0.00641 (8)	0.10233 (4)	0.0694 (2)	
S6	0.67818 (6)	−0.28177 (8)	0.11090 (4)	0.0738 (2)	
N1	0.4118 (2)	−0.0919 (3)	0.23396 (12)	0.0877 (9)	
N2	0.4099 (2)	0.3649 (2)	0.05811 (9)	0.0601 (6)	
N3	0.8704 (2)	−0.2290 (3)	0.10423 (13)	0.0879 (9)	
C1	0.4483 (2)	−0.0902 (3)	0.18492 (12)	0.0648 (8)	
C2	0.3332 (4)	−0.1872 (4)	0.24476 (18)	0.1292 (19)	
H2A	0.3525	−0.2272	0.2815	0.155*	
H2B	0.3285	−0.2589	0.2172	0.155*	
C3	0.2298 (3)	−0.1208 (4)	0.24245 (16)	0.0900 (12)	
C4	0.1609 (4)	−0.1736 (4)	0.27471 (19)	0.1262 (18)	
H4	0.1788	−0.2466	0.2983	0.151*	
C5	0.0658 (4)	−0.1170 (5)	0.2714 (2)	0.1196 (18)	
H5	0.0189	−0.1533	0.2922	0.143*	
C6	0.0401 (4)	−0.0078 (6)	0.2379 (2)	0.1240 (17)	
H6	−0.0236	0.0314	0.2363	0.149*	
C7	0.1084 (3)	0.0430 (4)	0.20688 (19)	0.1088 (14)	
H7	0.0910	0.1174	0.1841	0.131*	
C8	0.2029 (3)	−0.0138 (4)	0.20872 (17)	0.0876 (11)	
H8	0.2484	0.0213	0.1868	0.105*	
C9	0.4454 (3)	0.0057 (6)	0.27812 (15)	0.150 (2)	
H9A	0.4107	−0.0106	0.3096	0.225*	
H9B	0.4303	0.0952	0.2642	0.225*	
H9C	0.5171	−0.0032	0.2896	0.225*	
C10	0.4463 (2)	0.2424 (3)	0.04907 (10)	0.0550 (7)	
C11	0.3016 (3)	0.3908 (3)	0.05803 (12)	0.0743 (10)	
H11A	0.2627	0.3175	0.0390	0.089*	
H11B	0.2828	0.4729	0.0372	0.089*	
C12	0.2743 (2)	0.4050 (3)	0.11678 (13)	0.0607 (8)	
C13	0.3135 (3)	0.3233 (3)	0.15949 (14)	0.0784 (10)	
H13	0.3579	0.2548	0.1530	0.094*	
C14	0.2885 (3)	0.3406 (4)	0.21247 (15)	0.0838 (10)	
H14	0.3167	0.2842	0.2414	0.101*	
C15	0.2231 (3)	0.4387 (4)	0.22273 (16)	0.0859 (11)	
H15	0.2061	0.4501	0.2584	0.103*	
C16	0.1829 (3)	0.5200 (4)	0.17998 (19)	0.1046 (14)	
H16	0.1376	0.5874	0.1864	0.126*	
C17	0.2083 (3)	0.5040 (4)	0.12737 (16)	0.0862 (11)	
H17	0.1804	0.5610	0.0986	0.103*	
C18	0.4749 (3)	0.4831 (3)	0.07046 (13)	0.0810 (11)	
H18A	0.4547	0.5315	0.1014	0.122*	
H18B	0.4686	0.5406	0.0382	0.122*	
H18C	0.5442	0.4548	0.0799	0.122*	
C19	0.7783 (2)	−0.1785 (3)	0.10589 (12)	0.0619 (8)	
C20	0.9589 (3)	−0.1455 (4)	0.0978 (2)	0.1137 (15)	
H20A	1.0093	−0.1554	0.1309	0.136*	
H20B	0.9383	−0.0517	0.0952	0.136*	
C21	1.0065 (3)	−0.1816 (4)	0.0468 (2)	0.0894 (11)	
C22	1.1097 (3)	−0.1881 (4)	0.0502 (2)	0.0982 (12)	
H22	1.1502	−0.1691	0.0841	0.118*	
C23	1.1541 (5)	−0.2218 (6)	0.0053 (3)	0.139 (2)	
H23	1.2244	−0.2251	0.0085	0.167*	
C24	1.0969 (7)	−0.2506 (6)	−0.0441 (3)	0.167 (3)	
H24	1.1272	−0.2774	−0.0746	0.201*	
C25	0.9920 (6)	−0.2399 (7)	−0.0489 (3)	0.191 (3)	
H25	0.9517	−0.2572	−0.0831	0.229*	
C26	0.9477 (4)	−0.2037 (6)	−0.0031 (3)	0.145 (2)	
H26	0.8777	−0.1945	−0.0063	0.174*	
C27	0.8893 (3)	−0.3733 (4)	0.1102 (2)	0.1227 (16)	
H27A	0.9593	−0.3914	0.1078	0.184*	
H27B	0.8470	−0.4206	0.0809	0.184*	
H27C	0.8743	−0.4029	0.1460	0.184*	

### Atomic displacement parameters (Å^2^)

**Table:**

	*U*^11^	*U*^22^	*U*^33^	*U*^12^	*U*^13^	*U*^23^
Tl1	0.05321 (7)	0.04376 (6)	0.05131 (6)	0.00237 (6)	0.01089 (4)	−0.00302 (6)
S1	0.0853 (6)	0.0551 (5)	0.0967 (6)	−0.0087 (4)	0.0411 (5)	−0.0095 (5)
S2	0.0672 (6)	0.0906 (7)	0.0610 (5)	−0.0032 (5)	0.0052 (4)	−0.0268 (4)
S3	0.0613 (5)	0.0560 (4)	0.0684 (5)	0.0026 (4)	0.0135 (4)	−0.0121 (4)
S4	0.0686 (6)	0.0494 (4)	0.1041 (7)	0.0008 (4)	0.0310 (5)	0.0039 (4)
S5	0.0575 (5)	0.0601 (5)	0.0907 (6)	−0.0056 (4)	0.0118 (4)	−0.0074 (4)
S6	0.0661 (6)	0.0595 (5)	0.0983 (6)	0.0029 (4)	0.0203 (5)	0.0230 (5)
N1	0.086 (2)	0.116 (3)	0.0671 (19)	0.0459 (19)	0.0313 (16)	0.0198 (18)
N2	0.084 (2)	0.0478 (15)	0.0493 (14)	0.0118 (14)	0.0120 (13)	−0.0068 (11)
N3	0.0580 (19)	0.076 (2)	0.127 (3)	0.0062 (16)	0.0076 (17)	−0.0008 (18)
C1	0.067 (2)	0.072 (2)	0.0577 (18)	0.0242 (16)	0.0172 (16)	0.0057 (15)
C2	0.171 (5)	0.106 (3)	0.135 (4)	0.058 (3)	0.105 (4)	0.064 (3)
C3	0.113 (3)	0.065 (2)	0.109 (3)	0.006 (2)	0.072 (3)	0.000 (2)
C4	0.169 (5)	0.081 (3)	0.155 (4)	−0.001 (3)	0.114 (4)	−0.009 (3)
C5	0.132 (4)	0.097 (3)	0.152 (4)	−0.032 (3)	0.094 (4)	−0.027 (3)
C6	0.083 (3)	0.144 (4)	0.153 (5)	−0.022 (3)	0.047 (3)	−0.034 (4)
C7	0.081 (3)	0.115 (4)	0.133 (4)	0.005 (3)	0.025 (3)	0.002 (3)
C8	0.077 (3)	0.079 (3)	0.114 (3)	0.002 (2)	0.040 (2)	0.002 (2)
C9	0.117 (4)	0.282 (6)	0.050 (2)	0.074 (4)	0.009 (2)	−0.038 (3)
C10	0.073 (2)	0.0519 (17)	0.0401 (15)	0.0085 (15)	0.0079 (14)	−0.0025 (13)
C11	0.087 (3)	0.069 (2)	0.063 (2)	0.0315 (19)	0.0023 (18)	−0.0057 (16)
C12	0.062 (2)	0.0532 (18)	0.066 (2)	0.0076 (15)	0.0068 (16)	−0.0115 (15)
C13	0.103 (3)	0.063 (2)	0.070 (2)	0.025 (2)	0.016 (2)	−0.0013 (18)
C14	0.102 (3)	0.081 (3)	0.069 (2)	−0.004 (2)	0.017 (2)	0.007 (2)
C15	0.069 (2)	0.114 (3)	0.080 (2)	−0.009 (2)	0.027 (2)	−0.019 (3)
C16	0.089 (3)	0.126 (4)	0.105 (3)	0.039 (3)	0.036 (3)	−0.017 (3)
C17	0.092 (3)	0.086 (2)	0.083 (3)	0.035 (2)	0.020 (2)	−0.002 (2)
C18	0.133 (3)	0.0469 (18)	0.068 (2)	−0.003 (2)	0.033 (2)	−0.0070 (16)
C19	0.0542 (19)	0.071 (2)	0.0594 (18)	0.0083 (17)	0.0047 (15)	0.0032 (16)
C20	0.052 (2)	0.113 (3)	0.175 (4)	−0.002 (2)	0.012 (3)	−0.035 (3)
C21	0.064 (3)	0.073 (2)	0.132 (4)	−0.005 (2)	0.014 (3)	0.002 (2)
C22	0.064 (3)	0.095 (3)	0.137 (4)	0.006 (2)	0.021 (3)	0.022 (3)
C23	0.133 (5)	0.124 (4)	0.179 (6)	0.011 (4)	0.085 (5)	0.043 (4)
C24	0.238 (9)	0.129 (5)	0.161 (6)	−0.053 (6)	0.116 (7)	0.003 (5)
C25	0.223 (9)	0.207 (7)	0.145 (6)	−0.120 (7)	0.039 (6)	0.001 (5)
C26	0.102 (4)	0.170 (5)	0.158 (5)	−0.049 (4)	0.006 (4)	0.008 (4)
C27	0.093 (3)	0.086 (3)	0.188 (5)	0.038 (2)	0.017 (3)	0.032 (3)

### Geometric parameters (Å, °)

**Table:**

Tl1—S1	2.9241 (8)	C9—H9A	0.9600
Tl1—S2	2.6210 (8)	C9—H9B	0.9600
Tl1—S3	3.0242 (8)	C9—H9C	0.9600
Tl1—S4	2.8736 (8)	C11—C12	1.522 (4)
Tl1—S5	2.7325 (9)	C11—H11A	0.9700
Tl1—S6	2.8109 (8)	C11—H11B	0.9700
Tl1—S3^i^	3.1605 (8)	C12—C13	1.354 (4)
S1—C1	1.692 (3)	C12—C17	1.369 (4)
S2—C1	1.732 (3)	C13—C14	1.379 (4)
S3—C10	1.719 (3)	C13—H13	0.9300
S3—Tl1^i^	3.1605 (8)	C14—C15	1.354 (4)
S4—C10	1.714 (3)	C14—H14	0.9300
S5—C19	1.727 (3)	C15—C16	1.356 (5)
S6—C19	1.702 (3)	C15—H15	0.9300
N1—C1	1.345 (4)	C16—C17	1.371 (5)
N1—C9	1.461 (5)	C16—H16	0.9300
N1—C2	1.463 (5)	C17—H17	0.9300
N2—C10	1.339 (3)	C18—H18A	0.9600
N2—C18	1.463 (4)	C18—H18B	0.9600
N2—C11	1.466 (4)	C18—H18C	0.9600
N3—C19	1.332 (4)	C20—C21	1.510 (5)
N3—C27	1.459 (4)	C20—H20A	0.9700
N3—C20	1.469 (4)	C20—H20B	0.9700
C2—C3	1.522 (5)	C21—C26	1.352 (6)
C2—H2A	0.9700	C21—C22	1.368 (5)
C2—H2B	0.9700	C22—C23	1.351 (6)
C3—C8	1.354 (5)	C22—H22	0.9300
C3—C4	1.392 (5)	C23—C24	1.347 (7)
C4—C5	1.377 (6)	C23—H23	0.9300
C4—H4	0.9300	C24—C25	1.390 (8)
C5—C6	1.366 (6)	C24—H24	0.9300
C5—H5	0.9300	C25—C26	1.377 (7)
C6—C7	1.360 (5)	C25—H25	0.9300
C6—H6	0.9300	C26—H26	0.9300
C7—C8	1.374 (5)	C27—H27A	0.9600
C7—H7	0.9300	C27—H27B	0.9600
C8—H8	0.9300	C27—H27C	0.9600
			
S1—Tl1—S3	78.22 (2)	H9A—C9—H9C	109.5
S4—Tl1—S3	60.32 (2)	H9B—C9—H9C	109.5
S5—Tl1—S4	76.80 (3)	N2—C10—S4	120.1 (2)
S5—Tl1—S6	64.52 (3)	N2—C10—S3	120.3 (2)
S6—Tl1—S1	82.94 (2)	S4—C10—S3	119.54 (16)
S2—Tl1—S3^i^	163.05 (2)	N2—C11—C12	112.7 (2)
S2—Tl1—S5	86.57 (3)	N2—C11—H11A	109.0
S2—Tl1—S6	95.94 (3)	C12—C11—H11A	109.0
S2—Tl1—S4	86.63 (3)	N2—C11—H11B	109.0
S6—Tl1—S4	140.94 (2)	C12—C11—H11B	109.0
S2—Tl1—S1	64.44 (3)	H11A—C11—H11B	107.8
S5—Tl1—S1	134.02 (3)	C13—C12—C17	118.3 (3)
S4—Tl1—S1	131.55 (2)	C13—C12—C11	122.4 (3)
S2—Tl1—S3	84.84 (2)	C17—C12—C11	119.3 (3)
S5—Tl1—S3	136.63 (2)	C12—C13—C14	120.8 (3)
S6—Tl1—S3	158.72 (2)	C12—C13—H13	119.6
S5—Tl1—S3^i^	78.10 (2)	C14—C13—H13	119.6
S6—Tl1—S3^i^	84.05 (2)	C15—C14—C13	120.7 (3)
S4—Tl1—S3^i^	82.89 (2)	C15—C14—H14	119.7
S1—Tl1—S3^i^	132.08 (2)	C13—C14—H14	119.7
S3—Tl1—S3^i^	101.319 (18)	C14—C15—C16	118.8 (3)
C1—S1—Tl1	83.15 (11)	C14—C15—H15	120.6
C1—S2—Tl1	92.29 (10)	C16—C15—H15	120.6
C10—S3—Tl1	84.77 (10)	C15—C16—C17	120.8 (4)
C10—S3—Tl1^i^	87.45 (9)	C15—C16—H16	119.6
Tl1—S3—Tl1^i^	78.681 (18)	C17—C16—H16	119.6
C10—S4—Tl1	89.75 (10)	C12—C17—C16	120.7 (4)
C19—S5—Tl1	87.73 (10)	C12—C17—H17	119.7
C19—S6—Tl1	85.68 (10)	C16—C17—H17	119.7
C1—N1—C9	121.5 (4)	N2—C18—H18A	109.5
C1—N1—C2	121.8 (3)	N2—C18—H18B	109.5
C9—N1—C2	116.7 (3)	H18A—C18—H18B	109.5
C10—N2—C18	122.9 (3)	N2—C18—H18C	109.5
C10—N2—C11	122.6 (3)	H18A—C18—H18C	109.5
C18—N2—C11	114.4 (2)	H18B—C18—H18C	109.5
C19—N3—C27	120.9 (3)	N3—C19—S6	120.8 (2)
C19—N3—C20	123.3 (3)	N3—C19—S5	119.9 (2)
C27—N3—C20	115.8 (3)	S6—C19—S5	119.32 (17)
N1—C1—S1	122.4 (3)	N3—C20—C21	113.2 (3)
N1—C1—S2	117.4 (3)	N3—C20—H20A	108.9
S1—C1—S2	120.11 (17)	C21—C20—H20A	108.9
N1—C2—C3	112.5 (3)	N3—C20—H20B	108.9
N1—C2—H2A	109.1	C21—C20—H20B	108.9
C3—C2—H2A	109.1	H20A—C20—H20B	107.8
N1—C2—H2B	109.1	C26—C21—C22	119.4 (5)
C3—C2—H2B	109.1	C26—C21—C20	120.4 (4)
H2A—C2—H2B	107.8	C22—C21—C20	120.2 (4)
C8—C3—C4	119.6 (4)	C23—C22—C21	121.2 (5)
C8—C3—C2	121.3 (3)	C23—C22—H22	119.4
C4—C3—C2	119.1 (4)	C21—C22—H22	119.4
C5—C4—C3	119.4 (4)	C24—C23—C22	120.3 (6)
C5—C4—H4	120.3	C24—C23—H23	119.8
C3—C4—H4	120.3	C22—C23—H23	119.8
C6—C5—C4	120.4 (4)	C23—C24—C25	119.2 (7)
C6—C5—H5	119.8	C23—C24—H24	120.4
C4—C5—H5	119.8	C25—C24—H24	120.4
C7—C6—C5	119.4 (5)	C26—C25—C24	119.9 (7)
C7—C6—H6	120.3	C26—C25—H25	120.1
C5—C6—H6	120.3	C24—C25—H25	120.1
C6—C7—C8	121.1 (5)	C21—C26—C25	119.8 (6)
C6—C7—H7	119.5	C21—C26—H26	120.1
C8—C7—H7	119.5	C25—C26—H26	120.1
C3—C8—C7	120.0 (4)	N3—C27—H27A	109.5
C3—C8—H8	120.0	N3—C27—H27B	109.5
C7—C8—H8	120.0	H27A—C27—H27B	109.5
N1—C9—H9A	109.5	N3—C27—H27C	109.5
N1—C9—H9B	109.5	H27A—C27—H27C	109.5
H9A—C9—H9B	109.5	H27B—C27—H27C	109.5
N1—C9—H9C	109.5		

Symmetry codes: (i) −*x*+1, −*y*, −*z*.

### Hydrogen-bond geometry (Å, °)

**Table:**

*D*—H···*A*	*D*—H	H···*A*	*D*···*A*	*D*—H···*A*
C2—H2B···S1	0.97	2.51	3.030 (5)	113
C11—H11A···S3	0.97	2.51	3.009 (3)	112
C18—H18C···S4	0.96	2.51	3.008 (3)	112
C20—H20B···S5	0.97	2.46	3.012 (4)	116
C18—H18A···S1^ii^	0.96	2.87	3.654 (3)	140

Symmetry codes: (ii) *x*, *y*+1, *z*.
